# Highly reliable GIGA-sized synthetic human therapeutic antibody library construction

**DOI:** 10.3389/fimmu.2023.1089395

**Published:** 2023-04-27

**Authors:** Chao-Yang Huang, Ying-Yung Lok, Chia-Hui Lin, Szu-Liang Lai, Yen-Yu Wu, Chih-Yung Hu, Chu-Bin Liao, Chen-Hsuan Ho, Yu-Ping Chou, Yi-Hsuan Hsu, Yu-Hsun Lo, Edward Chern

**Affiliations:** ^1^ niChe Lab for Stem Cell and Regenerative Medicine, Department of Biochemical Science and Technology, National Taiwan University, Taipei, Taiwan; ^2^ Development Center for Biotechnology, New Taipei City, Taiwan; ^3^ Research Center for Developmental Biology and Regenerative Medicine, National Taiwan University, Taipei, Taiwan

**Keywords:** phage display, single-chain variable fragment (scFv), synthetic antibody library, antibody therapeutics, TIM-3

## Abstract

**Background:**

Monoclonal antibodies (mAbs) and their derivatives are the fastest expanding category of pharmaceuticals. Efficient screening and generation of appropriate therapeutic human antibodies are important and urgent issues in the field of medicine. The successful *in vitro* biopanning method for antibody screening largely depends on the highly diverse, reliable and humanized CDR library. To rapidly obtain potent human antibodies, we designed and constructed a highly diverse synthetic human single-chain variable fragment (scFv) antibody library greater than a giga in size by phage display. Herein, the novel TIM-3-neutralizing antibodies with immunomodulatory functions derived from this library serve as an example to demonstrate the library’s potential for biomedical applications.

**Methods:**

The library was designed with high stability scaffolds and six complementarity determining regions (CDRs) tailored to mimic human composition. The engineered antibody sequences were optimized for codon usage and subjected to synthesis. The six CDRs with variable length CDR-H3s were individually subjected to β-lactamase selection and then recombined for library construction. Five therapeutic target antigens were used for human antibody generation *via* phage library biopanning. TIM-3 antibody activity was verified by immunoactivity assays.

**Results:**

We have designed and constructed a highly diverse synthetic human scFv library named DSyn-1 (DCB Synthetic-1) containing 2.5 × 10^10^ phage clones. Three selected TIM-3-recognizing antibodies DCBT3-4, DCBT3-19, and DCBT3-22 showed significant inhibition activity by TIM-3 reporter assays at nanomolar ranges and binding affinities in sub-nanomolar ranges. Furthermore, clone DCBT3-22 was exceptionally superior with good physicochemical property and a purity of more than 98% without aggregation.

**Conclusion:**

The promising results illustrate not only the potential of the DSyn-1 library for biomedical research applications, but also the therapeutic potential of the three novel fully human TIM-3-neutralizing antibodies.

## 1 Introduction

Therapeutic monoclonal antibodies (mAbs) have achieved remarkable success in clinical applications due to their exquisite specificity and high affinity for antigen targeting (1). As of November 2020, more than one hundred mAb-based drugs have been approved for treatment for various human diseases including cancer, autoimmune, infectious and metabolic diseases through different mechanisms (2). MAbs can not only bind to surface biomarker antigens but also block the interaction between ligand and receptor thereby inhibiting the physiological activity of tumor cells or agonistic antibodies to death receptors can induce tumor cell death. The clinical utilization of mAbs include targeting cells through antibody-dependent cellular cytotoxicity (ADCC) and complement-dependent cytotoxicity (CDC) pathways to kill target cells (3–5), and also generating warheads for antibody–drug conjugates (ADCs). Additionally, mAbs can aid in designing novel immunocytokines, immunotoxins, bispecific Abs, chimeric antigen receptor (CAR) T cells, intracellular antibodies, and agonistic antibodies for death receptors (5–9). Based on these different strategies, mAbs and their derivatives have become one of the most rapidly expanding class of pharmaceuticals (10, 11). However, development of a reliable and highly efficient process to generate and screen potent human antibodies still needs to be explored further.

MAbs from different species have been generated for clinical use including mice, rats, rabbits, sharks, and camalids. However, due to immunogenicity risk (12), human mAbs have become the mainstream of current therapeutic antibody development. Phage display, human immunoglobulin loci-transgenic mice, and single B cell technology are the three most widely used approaches for the generation of fully human antibodies (13). Compared to the time-consuming approach of obtaining mAbs from immunized mice, target-specific mAbs can be rapidly isolated within weeks by the phage display platform (14–16). Based on the fusion of antibody fragments to M13 filamentous bacteriophage envelope proteins (17), phage display libraries can generate tremendous diversity of antibody fragments on the phage surface (18, 19). With standard panning methodologies, up to hundreds of phage binders can be isolated. Phage display antibody libraries have proven to be a powerful and efficient tool to isolate diagnostic or potential therapeutic antibodies (18).

There are many types of phage display libraries such as natural, immune, semi-synthetic and synthetic; and they are classified by the source of their sequences (19). A natural library can be obtained from various human B-cells and are relatively similar to the natural human antibody repertoire. Immune libraries are constructed from infection-recovered humans or immunized animals and can yield high affinity binders against targets from a small sized immune library (~10^6^). The diversity of a semi-synthetic library is increased through mutating one or more CDRs by using oligonucleotide-directed mutagenesis or mixing synthetic and natural sequences (18, 19). A synthetic library is composed of artificially designed and synthesized antibody sequences (20, 21). Engineered semi-synthetic and synthetic libraries have several advantages including the use of scaffolds with highly stable properties and codon-optimized sequences with high expression levels (21, 22). Generally, immune libraries are directed towards limited number of targets. Natural, semi-synthetic or synthetic libraries can be used to isolate high affinity binders against broad antigens but are required to have very high diversity (~10^10^ to 10^11^).

The size and quality of diversity design and functional clones of the antibody library are crucial for the success of isolating potential therapeutic antibodies (20–22). A number of strategies have been undertaken to design and construct large antibody libraries with high functional diversity, which are comprised of the number of correctly assembled clones without frameshift or stop codons (23–25), such as by synthesizing CDR sequences employing the trinucleotide mutagenesis (TRIM) technology or selecting in-frame antibody clones by antibiotic selection systems (15, 19). The TRIM approach uses a set of pre-synthesized trinucleotide codon units instead of single nucleotides, thereby has the ability to synthesize desired compositions at each CDR found in natural human antibodies and avoid frameshifts or stop codons (26). To rapidly isolate human antibodies with therapeutic potential, we designed and constructed a highly diverse synthetic human scFv library with greater than a giga in size named DSyn-1. The library design was based on high stability scaffolds and six CDRs that were tailored to mimic natural human compositions for a lower risk of immunogenicity. In addition, to maximize the number of in-frame clones with high expression levels, the antibody sequences were codon-optimized and the six CDRs with various CDR-H3 lengths were subjected to β-lactamase selection. To evaluate the Dsyn-1 library, phage panning was performed against five therapeutically relevant antigens including one peptide. Furthermore, to evaluate the library’s potential for biomedical research applications, we generated antibodies targeting the T cell immunoglobulin-3 (TIM-3), which is a type I trans-membrane protein expressed on both adaptive and innate immune cells such as effector T cells, natural killer cells, dendritic cells, and monocytes (27, 28). TIM-3 acts as an immune checkpoint inhibitor associated with anti-tumor immunity, and blockade of TIM-3 by antibodies has emerged as a potential therapeutic strategy for cancer treatment (29–31). In our library system, 204 unique antibodies recognizing TIM-3 were isolated and 25 were characterized *via* binding assays and *in vitro* analyses for inhibition ability. Based on our results, three novel TIM-3-specific antibodies were obtained from the DSyn-1 library.

## 2 Materials and methods

### 2.1 Analysis and design of CDR sequences

The human antibody heavy chain variable region (VH) and light chain variable region (VL) sequences were obtained from the IGHV3-23 and IGKV1-39 domain for humanized 4D5 (32) and the likely antigen contact residues were chosen for generating library diversity based on public information (33, 34). The three-dimensional structure model of scFv template (VH-linker (Gly4Ser)3-VL) was generated by Accelrys Discovery Studio 2019 (Accelrys, San Diego, CA, USA) and indicated the CDR residues chosen for diversification. Variable region sequences of the human antibodies were collected from the DCB database (human antibody sequences from DCB naïve library). The variable region sequences were analyzed using DCB’s proprietary algorithm. The percentage of each of the 20 amino acids at each CDR position was tabulated and imported into Microsoft Excel for simulating the six CDRs.

### 2.2 Construction of the Dsyn-1 scFv phage display library

**Figure 1:**
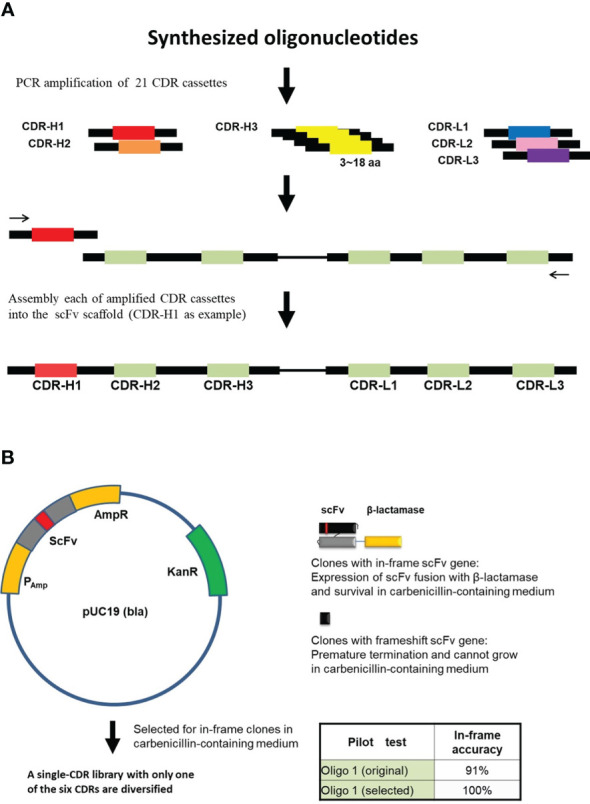
In-frame selection of assembled scFv repertoires. **(A)** Each of the CDR cassettes was amplified from synthesized oligonucleotides and then assembled into the scFv scaffold by PCR-based method (See “Materials and Methods”). Assembly of CDR H1 is used as an example. **(B)** In-frame selection system, proofreading of the assembled scFv sequences using the pUC19 (bla) vector. The assembled scFv repertoire was inserted between the β-lactamase signal sequence and the N-terminus of the β-Lactamase. A single-CDR library with only one of the six CDRs diversified was constructed for each CDR and selected for in-frame clones in carbenicillin-containing medium. In-frame selection of CDR cassettes were performed one by one. Oligo 1, one of the synthesized oligonucleotides.

**Figure 2:**
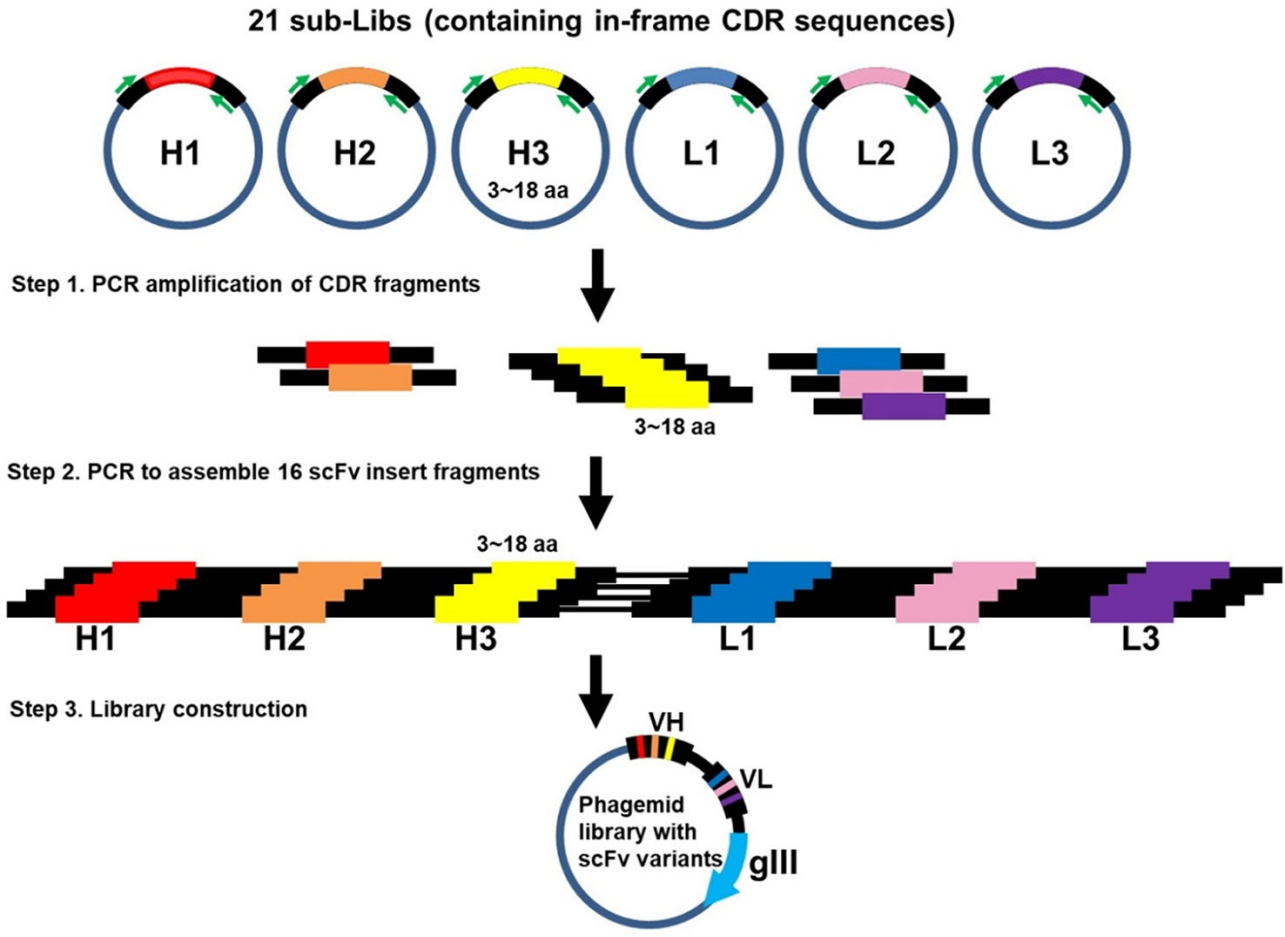
Construction of Dsyn-1 library. Several steps were implemented to generate the Dsyn-1 library. Step 1. CDR fragments were amplified by PCR from 21 sub-libraries (Libs), respectively. Step 2. 6 CDRs, CDRH1 to CDRL3, were assembled into a scFv insert fragment by using two-step overlap extension PCR. Each of the 16 scFv insert fragments was assembled according to different CDR-H3 lengths from 3 to 18 amino acids. Step 3. 16 scFv insert fragments were combined and cloned into phagemid.

The scFv template was codon-optimized and synthesized by GeneArt Gene Synthesis (Thermo Fisher Scientific, Waltham, MA, USA). CDR cassettes with both flanking sequences for polymerase chain reaction (PCR) were synthesized based on TRIM technology (Thermo Fisher Scientific). The CDR cassettes were amplified by PCR using flanking specific primers and inserted into the template scFv sequence by two-step overlap extension PCR (35) and cloned into pUC19-bla vector individually (**Figure 1**). Each ligation product was electroporated and selected for in-frame antibody clones in carbenicillin-containing 2YT medium. After carbenicillin medium selection, vectors containing proofreading CDR sequences were isolated. Six proofreading CDRs (three heavy chain CDRs and three light chain CDRs) were amplified and assembled into scFv fragments by PCR (**Figure 2**) and subsequently cloned into the pCANTAB 5E phagemid vector (Amersham Biosciences, Little Chalfont, Buckinghamshire, UK). The final ligation product was electroporated into *Escherichia coli* (E. coli) TG1 (Lucigen, Middleton, WI, USA).

### 2.3 Preparation of Dsyn-1 scFv phage clones

The TG1 library was inoculated into 2YT medium containing 100 μg/ml ampicillin and 2% glucose (2YT-AG) and grown to mid-log phase. The culture was then infected by incubation with Hyperphage (Progen, Heidelberg, Germany) or the M13KO7 helper phage (New England Biolabs, Hitchin, Hertfordshire, UK) at 37°C for 30 min without shaking. Infected cells were collected by centrifugation, resuspended in 2YT medium containing 100 μg/mL ampicillin and 25 μg/mL kanamycin (2YT-AK), and grown at 30°C overnight with shaking. Library phage particles were precipitated by PEG/NaCl (20% PEG 8000, 2.5 M NaCl) at 4°C for 1 h. After centrifugation, the pellet was resuspended in PBS. The scFv phage clones were subjected to three or four rounds of panning to select for specific antibodies.

### 2.4 Selection of Dsyn-1 scFv phage clones (solid-phase panning)

96-well MaxiSorp^®^ ELISA plate (Nunc, Rochester, NY, USA) was coated with human recombinant protein in 50 mmol/L sodium hydrogen carbonate (pH 9.6) at 4°C overnight and blocked with PBS containing 5% skim milk (MPBS), followed by washing with PBS. Phage particles (10^10^ to 10^11^ PFU) were incubated in antigen-coated wells at 37°C for 90 min and then washed with 0.05% Tween 20-PBS (PBST). The bound phages were eluted by adding triethylamine (100 mmol/L) with continuous rotation at 37°C for 30 min and neutralized with Tris buffer (1 mol/L, pH 7.4). The eluted phages were added to the exponentially growing TG1 bacteria and incubated for 30 min at 37°C without shaking for infection. The infected TG1 bacteria were collected by centrifugation, plated on 2YT-AG plates, and grown at 30°C overnight. Colonies grown on the plates were scraped using a glass spreader and grown to mid-log phase in 2YT-AG. The bacterial culture was infected with M13KO7 helper phage followed by the procedure described above to prepare scFv phage clones for the next round of selection with reduced concentrations of recombinant protein.

### 2.5 Selection of Dsyn-1 scFv phage clones (solution-phase panning)

To remove non-specific binders, the scFv phages (10^10^ to 10^11^ PFU) were pre-incubated with streptavidin-coated paramagnetic beads (Dynabeads M-280) (Invitrogen, Carlsbad, CA, USA) in 2% MPBS. The paramagnetic beads were removed and biotinylated human recombinant protein was added. The resultant mixture was rotated at 37°C for 90 min. Phages bound to biotinylated protein were captured by 2% MPBS-equilibrated Dynabeads. The beads were then washed with 0.05% PBST, 2% MPBS and PBS sequentially. Bound phages were eluted by triethylamine followed by the procedure described above to prepare scFv phages for the next round of selection.

### 2.6 Screening of positive phage binders by ELISA

Individual colonies randomly picked from an output plate of 3 or 4 rounds of selection were inoculated in 96-well plates and rescued with M13KO7 helper phage as described above for phage ELISA. ELISA plates were coated with 0.1 μg to 0.2 μg protein antigen per well. Wells were washed with PBS and blocked with 2% MPBS at 37°C for 2 h. Phage culture supernatant was added and incubated at 37°C for 90 min. The test solution was discarded and washed with PBST. An appropriate dilution of HRP-anti-M13 antibody (Sino Biological, Beijing, China) in 2% MPBS was added, incubated at 37°C for 90 min, washed with PBST, and developed with substrate solution 3,3’,5,5’-tetramethylbenzidine (KPL). Reaction was stopped by adding 1.0 M sulfuric acid and OD at 450 nm and at 650 nm was measured. Readings were obtained by subtracting OD 650 from OD 450.

### 2.7 Expression of full-length IgG antibodies

To produce full-length human IgG antibodies, the heavy and light chain variable regions obtained from phage panning were subcloned into expression vector as described previously (35). Transient transfection was performed in FreeStyle 293 or Expi 293 cells (Invitrogen). 37.5 µg of plasmid DNA and 75 µg of linear polyethyleneiminie (Polysciences, Warrington, PA, USA) were added in 150 mmol/L NaCl, respectively. DNA and PEI solutions were allowed to stand at room temperature for 5 min. The solutions were mixed gently and allowed to stand at room temperature for another 10 min. DNA-PEI mixture was added into 293 cells and incubated for 4 h. An equal volume of fresh culture medium was added and the cells were cultured for 5 to 7 days. Culture supernatant containing the antibody was affinity-purified using the Montage Antibody Purification kit (Millipore, Billerica, MA, USA).

### 2.8 Determination of antibody binding affinity by ELISA

Antigen coating conditions and washing stringency were as described in phage ELISA. Wells were blocked with 5% MPBS or 1% BSA in PBS at 37°C for 1 h and incubated with serial diluted antibodies for 1 h at 37°C. After washing, goat-anti-human kappa light chain-HRP (1:10,000) (Jackson ImmunoResearch,West Grove, PA, USA) was added into each well. Absorbance was measured as described above. The binding affinities of the antibodies were calculated using non-linear regression with Prism software (GraphPad, San Diego, CA, USA).

### 2.9 TIM-3 reporter assay

The biological activity of TIM-3-recognizing antibodies which can block TIM-3 signaling was measured by the TIM-3 reporter assay (Promega, Madison, WI, USA). Various concentrations of anti-TIM-3 antibodies or reference antibody 2E2, TIM-3 target and effector cells, were plated together in 96-well Tissue Culture Plates (TPP, Zollstrasse, Trasadingen, Switzerland). After 17 h induction at 37°C, Bio-Glo-NL™ Reagent was added and luminescence was measured by NanoLuc^®^ luminescence in a GloMax^®^ Discover Plate Reader. The inhibition ratio (%) was calculated by the formula: 100% – (RLU Ab dilution/RLU no antibody control) × 100%.

### 2.10 T cell proliferation assay

Blood sample was diluted at 1:1 with PBS. Peripheral blood mononuclear cells (PBMCs) were prepared from blood sample using Ficoll-Paque density gradient media (GE Healthcare Bio-Sciences Corp., Marlborough, MA, USA) according to the manufacturer’s protocol. After wash with PBS, CD3^+^ T cells were further isolated from PBMCs by MACS human CD3 MicroBeads kit (Miltenyi Biotec, Bergisch Gladbach, Germany). Non-treatment CD3^+^ T cells were plated at the density of 1 × 10^5^ cells/well in 96 well plates. Activation of CD3^+^ T Cells was performed by Dynabeads Human T-activator CD3/28 (Invitrogen) with cells to beads ratio 1:1. After activation for 2 days, the Dynabeads were removed by placing the tube on a magnet for 1-2 min. Activated CD3^+^ T cells were plated at the density of 1 × 10^5^ cells/well in 96 well plates and anti-TIM3 antibody, reference antibody MBG-453 (sequence obtained from the immunogenetics (IMGT) database (http://imgt.org) or Human IgG4 were added. After 1 h, galectin-9 was added. To determine T cell proliferation the DELFIA assay was performed according to the manufacturer’s instructions from PerkinElmer (Milan, Italy). Measure the Eu-fluorescence in a time-resolved fluorometer (TRF).

### 2.11 Antibody analysis by size exclusion chromatography and high performance liquid chromatography

Analysis was performed on an Agilent 1260 isocratic pump HPLC system equipped with an Agilent AdvanceBio SEC 300Å column (Agilent, Santa Clara, CA, USA). Protein samples at 2 mg/mL were injected for analysis. Isocratic flow of a mobile phase consisting of 0.1 M sodium phosphate, 0.1 M sodium sulfate and 0.02% sodium azide (pH 6.8) was at 0.6 mL/min for 26 min. Separation was monitored by UV absorption at 280 nm.

## 3 Results

### 3.1 Design of the highly diverse synthetic human scFv library

**Figure 3:**
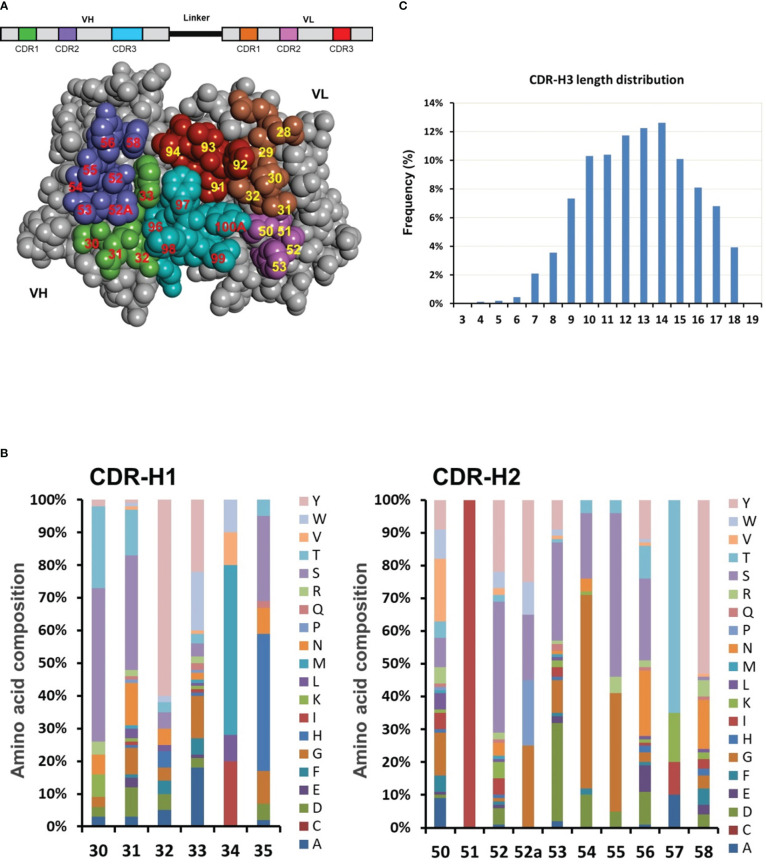
Dsyn-1 library design. **(A)** The library is based on the VH-linker-VL (scFv) format and the three-dimensional structure of VH and VL domains are shown as a space-filling model. The potential antigen contact residues in the CDRs chosen for diversification are colored green (CDR-H1), purple (CDR-H2), cyan (CDR-H3), brown (CDR-L1), pink (CDR-L2) or red (CDR-L3), and numbered according to the Kabat nomenclature. This Figure was generated with Discovery Studio Visualizer 2019 (Dassault Systèmes BIOVIA, San Diego) using coordinates for the crystal structure of free Fv4D5 (PDB entry 2FJF). **(B)** Engineered diversity of CDR-H1 and CDR-H2. The natural diversity was calculated from the alignment of thousands of antibody sequences from the DCB database for simulating six CDR variability. The designed percentage of each residue at each CDR position is schematically illustrated. **(C)** The designed CDR-H3 length distribution (Kabat’s definition) is based on natural length distribution.

To obtain highly diverse and functional human antibodies, we designed a synthetic human library named DSyn-1 in the scFv antibody fragment format. To construct highly reliable human antibody library as therapeutics, human IGHV3-23 (VH) and IGKV1-39 (VL) germline genes were chosen as the scaffolds for the scFv template, which were previously published to have higher thermal stability, higher expression yields, and displayed well on bacteriophage (33, 36). Based on the high-resolution IGHV3-23 and IGKV1-39 related structures available from the Protein Data Bank, the potential antigen contact residues on the six CDRs of the antibody were chosen for library design and shown on the three-dimensional structure model of VH and VL domains in **Figure 3A**. H30-35, H50-58 and H95-102 on the heavy chain variant region and L28-34, L50-53 and L89-96 on the light chain variant region were selected as the sites for the design of the library.

Thousands of human antibody sequences were collected from the DCB database for detailed analysis with focus on sequence variability in the six CDRs. The percentage of each of the 20 amino acids at each CDR position was tabulated (**Figure 3B**, schematically illustrated) and utilized to closely simulate the six CDR variability including the composition of different CDR-H3 lengths from 3 to 18 amino acids covering about 80% of the natural CDR-H3 length repertoire (**Figure 3C**) (37, 38). Diversified antigen contact positions at CDR-H1 and CDR-H2 were schematically illustrated as an example in **Figure 3B**.

ScFv fragments of phage display showed good expression in bacteria and human IgG antibody was well expressed in the mammalian expression system. The scFv template and engineered CDR cassette gene sequences were subsequently optimized to enhance antibody expression in both bacterial and mammalian expression systems. Subsequently the scFv template and each CDR cassette were subjected to oligonucleotide synthesis.

### 3.2 Construction of the Dsyn-1 library

In order to generate the Dsyn-1 library, several steps were implemented (**Figures 1**, **2**). First, 21 CDR cassettes, including five CDRs, CDR-H1, H2, L1, L2, L3, and 16 CDR-H3s with different lengths, were amplified from synthesized oligonucleotides and then each amplified CDR was assembled into scFv scaffold by PCR-based method resulting in 21 CDR cassettes including scFv insert fragments (**Figure 1A**, CDR-H1 as an example).

Next, to omit frameshift mutants in scFv insert fragments, we incorporated the β-lactamase (bla) in-frame selection system so that the *bla* gene was fused to the C-terminus of the assembled scFv gene in vector pUC19 to select the in-frame clones in carbenicillin medium (**Figure 1B**). For example, pilot test of oligo 1 showed that the ratio of in-frame clones was 91% before carbenicillin selection and 100% after proofreading selection.

The 21 CDR cassettes containing scFv insert fragments were cloned into vector pUC19 (bla) and subjected to in-frame selection one by one resulting in 21 sub-libraries to provide CDR repertoires for library construction (**Table 1**). The size of these sub-libraries ranged from 10^4^ to 10^9^ depending on the diversity of each CDR. The total number of colonies of the sub-libraries CDR-H1, H2, L1, L2, and L3 were more than 3.6 × 10^9^ in total and 16 CDR-H3 sub-libraries contained more than 2.6 × 10^10^ colonies, which were sufficient to generate a library with diversity size greater than 10^10^. After a series of amplification, assembly, and cloning, a sizable library was obtained with approximately 2.5 × 10^10^ colonies (**Figure 2**).

**Table 1:** Summary of in-frame selection results (21 sub-libraries (Lib)).

CDR	Design diversity	Size of sub-Lib	CDR	Design diversity	Size of sub-Lib
**H1**	1.43 x 10^6^	7.20 x 10^8^	**H3(9)**	3.67 x 10^8^	2.59 x 10^8^
**H2**	9.72 x 10^8^	1.83 x 10^9^	**H3(10)**	5.15 x 10^8^	1.82 x 10^8^
**L1**	2.15 x 10^6^	2.09 x 10^8^	**H3(11)**	5.20 x 10^8^	4.46 x 10^9^
**L2**	1.43 x 10^4^	5.04 x 10^5^	**H3(12)**	5.87 x 10^8^	2.20 x 10^9^
**L3**	3.57 x 10^8^	8.82 x 10^8^	**H3(13)**	6.13 x 10^8^	7.56 x 10^8^
**H3(3)** ^a^	6.86 x 10^3^	3.68 x 10^4^	**H3(14)**	6.31 x 10^8^	8.00 x 10^9^
**H3(4)**	1.30 x 10^5^	5.06 x 10^4^	**H3(15)**	5.05 x 10^8^	4.00 x 10^9^
**H3(5)**	2.48 x 10^6^	4.33 x 10^7^	**H3(16)**	4.05 x 10^8^	2.68 x 10^9^
**H3(6)**	2.24 x 10^7^	5.37 x 10^6^	**H3(17)**	3.40 x 10^8^	1.59 x 10^9^
**H3(7)**	1.05 x 10^8^	4.41 x 10^8^	**H3(18)**	1.96 x 10^8^	1.54 x 10^9^
**H3(8)**	1.77 x 10^8^	3.29 x 10^8^			

a H3(3): CDRH3 length 3 (length of 3 amino acids).

### 3.3 Sequence analysis of the Dsyn-1 library

**Figure 4:**
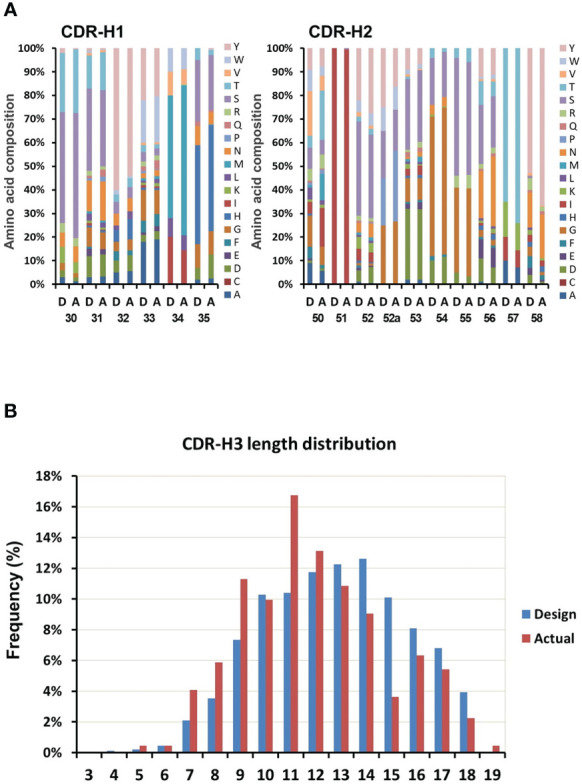
Variability assessment of the library. The variability in the designed library is compared with the variability found from sequencing 300 clones of the actual constructed library. **(A)** Comparison between designed (D, left columns) and actual (A, right columns) composition of CDRs (CDR1 and CDR2 as example). **(B)** The length distribution of the designed (D, left columns) and the actual (A, right columns) CDR-H3.

To assess the variability of the library, 300 randomly selected colonies were subjected to sequencing and analyzed for diversity of the six CDRs. Of these, 258 sequences were readable. Comparison of the amino acid composition to the actual composition of the constructed library indicated that the percentage of each of amino acid at each CDR position was similar. The composition of CDR-H1 and CDR-H2 was shown in **Figure 4A** as example. This revealed that gene synthesis, cloning, and antibiotic based proofreading did not affect the composition of the CDR residues. Distribution of CDR H3 length was shown in **Figure 4B**. The library showed coverage for most of the original CDR-H3 length distributions except the 3 and 4 amino acid length, as well as the 15 amino acid length which was conspicuously underrepresented in the design. Although sequence analysis did not reveal any clones with CDR-H3 lengths of 3 or 4 amino acids, they were however later found in following antigen selection experiments.

In order to maximize the size of in-frame clones, all different lengths CDR-H3s of the library were subjected to β-lactamase selection. After proofreading, sequence analysis of the CDR-H3 region from the library indicated that the frameshift mutation ratio located in this region was 2% (6 out of 258 readable sequences encoded frameshift mutations) lower than that of many public synthetic libraries that did not have proofread CDR-H3s (around 7 to 20% frameshift mutation rate) (15, 39).

### 3.4 Panning and screening of mAbs from Dsyn-1 library

Exploratory selections were performed to evaluate the potential of the Dsyn-1 library. The scFv phage library was panned against five therapeutically relevant antigens including TIM-3, TGF-β, B7H3, bacteria recombinant protein BA, and CCR5-peptide using solid-phase (immunoplate) or solution-phase (Dynabeads) panning methods.

After four rounds of panning against human TIM-3 recombinant protein, a total 1632 output colonies were randomly picked from the third and fourth round in phage ELISA screening. ELISA results showed the hit rate to be around 47% (772/1632). 772 clones with positive signals were sequenced to determine their VH and VL nucleotide sequences. A total of 204 unique scFv phage binders recognizing TIM-3 were obtained.

In the case of human TGF-β recombinant protein, after four rounds of panning, the ELISA results showed the hit rate to be around 95% (91/96). 91 clones with positive signals were sequenced and a total of 29 unique scFv phage binders recognizing protein TGF-β were obtained. The same procedure was used for B7H3 and bacteria recombinant protein BA to obtain 23 and 26 unique scFv phage binders and a 70% (134/192) and 86% (166/192) hit rate in the ELAISA assay, respectively. Additionally, we also evaluated the possibility of selecting for binders against peptide. CCR5-peptide was subjected to our biopanning system as antigen. The phage ELISA results showed 22% (237/1056) hit rate and 45 unique scFv phage binders were obtained. The selection results for each antigen are summarized in **Table 2**. Phage ELISA screening indicated hit rates ranging from 22–95% and sequencing of positive clones revealed 23 to 204 unique scFvs per antigen. The phage ELISA binding signal and specificity against TIM-3、TGF-β and CCR5-peptide are showed in **Tables S1-S3**.

**Table 2:** Summary of panning results.

No.	Antigen	Species	Phage ELISA (positive/screened)	Hit rate^b^	Unique clones
1	Protein (TIM3)	Human	772/1632	47%	**204**
2	Protein (TGF-β)	Human	91/96	95%	**29**
3	Protein (B7H3)	Human	134/192	70%	**23**
4	Peptide (CCR5)	Human	237/1056	22%	**45**
5	Protein (BA)^a^	Bacterium	166/192	86%	**26**

a Protein (BA): Bacterium recombinant protein, undisclosed target.

b Defined as number of positive clones divided by the number of screened clones.

### 3.5 Dsyn-1 library with the potential for biomedical applications

**Figure 5:**
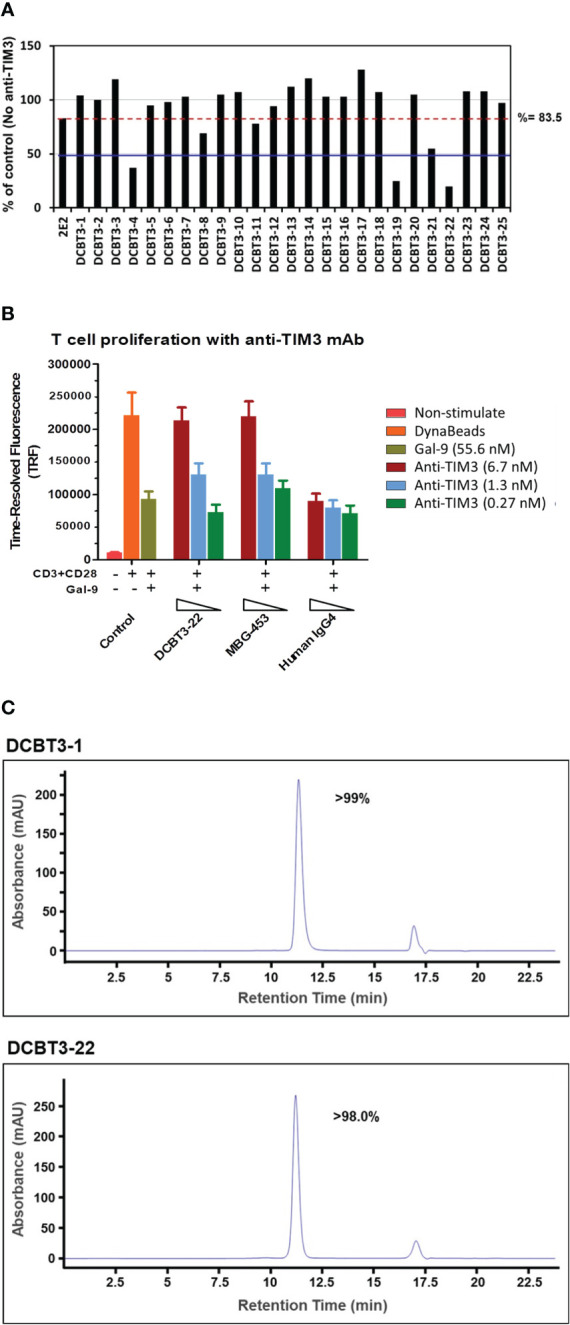
Characterization of purified TIM-3 antibodies. **(A)** The biological activity of TIM-3-recognizing antibodies and reference antibody 2E2 which can block the interactions between TIM-3 and it’s receptor in TIM-3 reporter assay at 1.11 mg/ml (7.3 nM). Red dotted line indicates the inhibition activity of reference antibody 2E2 at 7.3 nM. Blue full line indicates the 50% inhibition ratio at 7.3 nM. Percent of Control = (RLU Ab dilution/RLU no antibody control) × 100%. The inhibition ratio (%) was calculated by the formula: 100% – (RLU Ab dilution/RLU no antibody control) × 100%. **(B)** The biological activity of DCBT3-22 and reference antibody MBG-453 which can relieve the cell proliferation inhibitory effect of Galectin-9 ligand. No antibody treatment groups are as control. **(C)** SEC-HPLC results of purified TIM-3 antibodies. Analytical size-exclusion chromatogram of purified TIM-3 antibodies.

TIM-3 acts as an immune checkpoint inhibitor and blockade of TIM-3 by antibodies has emerged as a potential therapeutic strategy in cancer treatment. To demonstrate the library’s potential for pharmaceutical applications, 25 out of 204 unique antibodies recognizing TIM-3 were converted into full length IgGs for ELISA binding affinity analysis. The transient expression level of these antibodies were 0.23 to 6.06 mg/L in a 30 ml volume and their binding affinities (K_D_ values) were determined to be ranging from 0.9 μM to 0.06 nM. To assess the capability of inhibiting TIM-3 signaling induced promoter-mediated luminescence, all of the antibodies were subjected to TIM-3 reporter assay. As shown in **Fig 5A**, the inhibition rate of DCBT3-4, DCBT3-19 and DCBT3-22 is significantly superior to the reference antibody 2E2, at antibody concentration of 7.3 nM. The inhibition efficiency of DCBT3-4, DCBT3-19, DCBT3-22, and 2E2 were 63%, 75%, 80%, and 16.5%, respectively. Therefore, the EC_50_ of DCBT3-4, DCBT3-19 and DCBT3-22 was lower than 7.3 nM. In addition, the binding affinities of these three antibodies were 0.1 to 0.2 nM, satisfying one of the criteria for drug development.

To further evaluate the biological activity of superior clone DCBT3-22, a novel T cell proliferation assay was performed. The T cell proliferation assay is designed based on the biological function characteristics of the TIM-3/Galectin-9 signaling pathway, blocking the TIM-3/Galectin-9 signaling pathway resulting in relieving the cell proliferation inhibitory effect of Galectin-9 ligand. The experimental results are shown in **Figure 5B**. In control groups, compared with non-activated T cells (red bar, non-stimulated), the TRF intensity of activated T cells significantly increase (orange bar), but when the treatment of Galectin-9 at the concentration of 55.6 nM, the TRF intensity reduce to about 50%. In antibody treatment groups, antibodies were used to test at the concentrations of 0.27, 1.3 and 6.7 nM. The results indicate that DCBT3-22 and MBG-453 could relieve the cell proliferation inhibitory effect of Galectin-9 ligand at the concentration of 1.3 and 6.7 nM, the TRF intensity significant restoration, whereas Human IgG4 had no relieving effects.

Beyond the strong binding affinity and inhibition ability, antibody solubility (without aggregation) is the subsequent issue for the later stages of large-scale production. To further evaluate the physico-chemical property of the antibodies, SEC-HPLC was used for analysis (DCBT3-1 and DCBT3-22 were selected as example). DCBT3-22 showed potent inhibition activity against TIM-3 signaling, whereas DCBT3-1 had no inhibitory effects. After the optimization of transient transfection, the expression level of DCBT3-1 and DCBT3-22 were more than 10 mg/L. For SEC-HPLC analysis, the purity of DCBT3-1 was more than 99% and DCBT3-22 was more than 98% without aggregation or fragment production (**Figure 5C**).

Taken together, DCBT3-4, DCBT3-19 and DCBT3-22 derived from the Dsyn-1 library showed strong binding affinities in the sub-nanomolar range and significant inhibition activity against TIM-3 signaling within the nanomolar range. Moreover, the best clone DCBT3-22 demonstrated good expression level and physico-chemical property without aggregation.

## 4 Discussion

To date, more than one hundred mAb drugs have been granted approvals for the treatment of various human diseases (2). Following the success of these approvals, it has been realized that the desired affinity, specificity, and biological activity are not enough. Other biophysical properties such as expression level, stability and solubility as well as immunogenicity should also be considered in the early discovery stages of drug development (19, 22, 39, 40). These issues may be alleviated by the synthetic antibody generation strategy. The synthetic generation approach has several unique advantages. First, the nucleotide and amino acid contents of the antibody library can be fully controlled. Second, the library is designed based on high thermal stability scaffolds. Third, six CDRs can be designed with high diversity. Fourth, the engineered antibody sequences can be codon-optimized to exhibit high levels of expression in both prokaryotic and eukaryotic expression systems. In this study, we have designed and constructed a highly functional human antibody library containing approximately 2.5 × 10^10^ colonies based on the synthetic antibody approach while keeping the advantages described above. In addition, the six CDRs of the library are tailored to mimic the human composition of antibodies that has a lower risk of immunogenicity. In order to maximize the proportion of in-frame clones, the engineered antibody sequences were synthesized based on TRIM technology and all of the CDRs were subjected to β-lactamase selection. Furthermore, the promising results of generating TIM-3 neutralizing antibodies as described above demonstrate the potential of this library. Therefore, the size, diversity and quality of this library can probably be a reliable source of human antibodies for diagnostic and clinical applications.

All of the six CDRs in Dsyn-1 library including varying CDR-H3 lengths were subjected to β-lactamase selection resulting in total twenty one sub-libraries to offer six CDR repertoires of synthetic antibodies, which was distinguished from other known synthetic libraries whose five CDRs except the highly diverse CDR-H3 were subjected to the proofreading process. Since CDR-H3 loops are highly diverse and frequently contact with antigen (1), these loops lacking proofreading have frequently been reported to retain numerous unwanted clones in the library (14). In a previous study, the number of transformants (~10^7^) was not sufficient to encompass CDR-H3 diversity which led to this region not being submitted to the proofreading process. In this study, a tremendous effort was placed on proofreading all CDR-H3s with varying lengths to generate 16 sub-libraries (size from 3.68 × 10^4^ to 8.0 × 10^10^). In total, more than 2.6 × 10^10^ colonies of the 16 CDR-H3 sub-libraries were able to generate sufficient diversity for the construction of a 2.5 × 10^10^ library. Results from sequence analysis indicate that close to 2% of the frameshift mutations were located in the CDR-H3, and the nearly 2% of the frameshift mutations in Dsyn-1 library is much lower than that in other public synthetic libraries without proofreading of the CDR-H3 region (around 7-20% frameshift mutation rate) (14, 39). DSyn-1 is one of few synthetic libraries where six CDRs were subjected to proofreading individually and then recombined to form scFv fragments. Our results also confirm that even after proofreading by the β-lactamase selection, some clones still contain frameshifts in selected regions, consistent with the previous study (14).

The Dsyn-1 library was designed based on high stability scaffolds and six complementarity determining regions (CDRs) tailored to mimic natural composition. In our library, the oligo-nucleotides of six CDRs were synthesized based on TRIM technology. There are two types of methods for CDR diversification: degenerate nucleotide codons and TRIM technology. Degenerate codon method is the synthesis of random sequences using nucleotide mixtures (22). Commonly used degenerate codons in CDR design are NNK or NNS (N = A, T, C or G; K= G or T; S = G or C) that encodes all 20 amino acids. This approach is the simplest method and the most cost-efficient, but the major limitation is that the ratio of 20 amino acids can’t be adjusted to closely simulate the natural composition. In contrast, the TRIM approach utilizes a set of pre-synthesized trinucleotide codons that include the codons of all 20 amino acids for the synthesis of diversified CDRs, which can introduce a desired ratio of 20 amino acids at any position, thereby has the ability to synthesize desired compositions at each CDR.

To further assess the potential of the Dsyn-1 library, phage panning was performed against therapeutically relevant antigens such as recombinant proteins or peptides. After three to four rounds of selection, ELISA screening showed hit rates ranging from 21–95%, and sequencing of positive clones gave rise to 23 to 204 unique scFvs per antigen. These results indicated that the Dsyn-1 library has the potential to generate up to hundreds of specific binders to recognize each target.

CAT, Dyax and MorphoSys libraries are excellent phage display libraries published in the last decade and they are naïve, semi-synthetic and synthetic libraries, respectively (19). These libraries have the potential to generate up to hundreds of specific and high affinity (sub-nanomolar range) binders to recognize the targets of interest. Compared to these libraries, our Dsyn-1 library has the same efficiency, if not more, in generating high diversity and high affinity binders.

TIM-3 acts as a negative regulatory checkpoint molecule associated with both innate and adaptive immunity. Many mAbs have been developed based on the theory that inhibition of TIM-3 signaling can demonstrate therapeutic benefit for cancer immunotherapy. Some of these therapeutic antibodies include MBG453 (Novartis), TSR-022 (Tesaro) and LY3321367 (Eli Lilly). The blockade of TIM-3 signaling by antibodies has emerged as a potential immune checkpoint target for cancer treatment (29, 41). In this study, we have generated three TIM-3-blocking antibodies (DCBT3-4, DCBT3-19, and DCBT3-22) and demonstrated their ability to inhibit signaling-induced and promoter-mediated luminescence. All three antibodies showed significant inhibition of TIM-3 signaling in TIM-3 reporter assays at the nanomolar range and had binding affinities in the sub-nanomolar range. These three fully human antibodies have the potential to be developed as TIM-3 targeting agents for the treatment of cancer. In addition, in order to develop DCBT3-4, DCBT3-19 and DCBT3-22 for clinical applications, T cell activation assays and animal studies would need to be performed to further confirm their efficacy.

## 5 Conclusions

To rapidly isolate human antibodies with therapeutic potential, we designed and constructed a highly diverse synthetic human scFv library with thermal stability scaffolds and all of the CDRs were subjected to β-lactamase selection. Phage library panning revealed that the library has the potential to generate up to hundreds of specific binders recognizing each target. Furthermore, three TIM-3-blocking antibodies (DCBT3-4, DCBT3-19, and DCBT3-22) derived from the DSyn-1 library showed strong binding to human TIM-3 recombinant protein in the sub-nanomolar range and can significantly inhibit TIM-3 signaling in TIM-3 reporter assay in the nanomolar range. These promising results not only demonstrate the performance of DSyn-1 library for biomedical research development but also the potential of the three novel TIM-3-blocking antibodies for further clinical therapeutic application.

## Data availability statement

The original contributions presented in the study are included in the article/**Supplementary Material**. Further inquiries can be directed to the corresponding author.

## Author contributions

C-YaH, C-BL and C-HH conceived and designed the experiments. C-YaH, Y-YL, C-HL, S-LL, Y-YW, C-YuH, Y-PC and Yi-HH performed the experiments. C-YaH, Y-YL, C-HL, S-LL and Y-PC analyzed the data. C-YaH wrote the manuscript. Y-HL and EC supervised the study, and edited the manuscript. All authors contributed to the article and approved the submitted version.
