# Supplementary material for: Highly reliable GIGA-sized synthetic human therapeutic antibody library construction
Source: Front Immunol. 2023 Apr 27;14:1089395. doi: 10.3389/fimmu.2023.1089395 (PMC10174300; doi:10.3389/fimmu.2023.1089395)
Supplement: Supplementary file 1 [file Table_1.docx]

**Table S1. Phage ELISA against human TIM-3 recombinant protein**

| **Clone name** | **TIM3** | **hFc-cMyc-His^a^** | **Ratio^b^** |
| --- | --- | --- | --- |
| **DS3-1** | 2.54 | 0.064 | **39.7** |
| **DS3-2** | 2.203 | 0.126 | **17.5** |
| **DS3-3** | 2.613 | 0.055 | **47.5** |
| **DS3-4** | 2.49 | 0.081 | **30.7** |
| **DS3-6** | 2.253 | 0.066 | **34.1** |
| **DS3-7** | 1.949 | 0.088 | **22.1** |
| **DS3-8** | 2.587 | 0.107 | **24.2** |
| **DS3-12** | 2.051 | 0.117 | **17.5** |
| **DS3-13** | 2.495 | 0.077 | **32.4** |
| **DS3-14** | 2.289 | 0.049 | **46.7** |
| **DS3-15** | 1.635 | 0.062 | **26.4** |
| **DS3-16** | 2.347 | 0.155 | **15.1** |
| **DS3-17** | 2.944 | 0.263 | **11.2** |
| **DS3-18** | 2.378 | 0.111 | **21.4** |
| **DS3-22** | 2.227 | 0.047 | **47.4** |
| **DS3-25** | 2.292 | 0.098 | **23.4** |
| **DS3-27** | 2.053 | 0.181 | **11.3** |
| **DS3-28** | 2.67 | 0.132 | **20.2** |
| **DS3-33** | 2.574 | 0.123 | **20.9** |
| **DS3-34** | 2.568 | 0.05 | **51.4** |
| **DS3-40** | 2.566 | 0.047 | **54.6** |

^a^**hFc-cMyc-His:** Human IgG1 Fc-cMyc-His tag, as background control

^b^Defined as TIM-3 signal divided by hFc-cMyc-His signal

**Table S2 Phage ELISA against human TGF**-β **recombinant protein**

| **Clone name** | **TGF-beta** | **hFc-cMyc-His^a^** | **Ratio^b^** |
| --- | --- | --- | --- |
| **TGFb-2** | 3.377 | 0.638 | **5.3** |
| **TGFb-8** | 3.194 | 0.197 | **16.2** |
| **TGFb-9** | 3.285 | 0.129 | **25.5** |
| **TGFb-10** | 3.153 | 0.049 | **64.3** |
| **TGFb-13** | 0.869 | 0.142 | **6.1** |
| **TGFb-23** | 3.232 | 0.409 | **7.9** |
| **TGFb-24** | 3.193 | 0.263 | **12.1** |
| **TGFb-25** | 2.909 | 0.25 | **11.6** |
| **TGFb-34** | 3.152 | 0.611 | **5.2** |
| **TGFb-35** | 2.66 | 0.34 | **7.8** |
| **TGFb-36** | 0.989 | 0.105 | **9.4** |
| **TGFb-45** | 1.18 | 0.087 | **13.6** |
| **TGFb-46** | 1.602 | 0.116 | **13.8** |
| **TGFb-PB1** | 1.893 | 0.144 | **13.1** |
| **TGFb-PB6** | 3.181 | 0.266 | **12.0** |
| **TGFb-PB10** | 1.444 | 0.085 | **17.0** |
| **TGFb-PB11** | 2.224 | 0.071 | **31.3** |
| **TGFb-PB14** | 3.043 | 0.243 | **12.5** |
| **TGFb-PB18** | 2.498 | 0.19 | **13.1** |
| **TGFb-PB19** | 1.189 | 0.074 | **16.1** |
| **TGFb-PB21** | 3.311 | 0.094 | **35.2** |
| **TGFb-PB26** | 3.02 | 0.337 | **9.0** |
| **TGFb-PB27** | 2.805 | 0.202 | **13.9** |
| **TGFb-PB31** | 3.148 | 0.227 | **13.9** |
| **TGFb-PB32** | 2.914 | 0.374 | **7.8** |
| **TGFb-PB36** | 3.31 | 0.423 | **7.8** |
| **TGFb-PB39** | 2.86 | 0.127 | **22.5** |
| **TGFb-PB41** | 3.176 | 0.365 | **8.7** |
| **TGFb-PB46** | 1.868 | 0.141 | **13.2** |

^a^**hFc-cMyc-His,** as background control

^b^Defined as **TGF**-βsignal divided by hFc-cMyc-His signal

**Table S3 Phage ELISA against human CCR5-peptide**

| **Clone name** | **CCR5 peptide-Albumin** | **Albumin**^a^ | **Ratio**^b^ |
| --- | --- | --- | --- |
| **CCR5-H3-5** | 1.798 | 0.436 | **4.12** |
| **CCR5-H3-7** | 0.622 | 0.094 | **6.62** |
| **CCR5-H3-8** | 0.82 | 0.051 | **16.08** |
| **CCR5-H3-16** | 2.015 | 0.078 | **25.83** |
| **CCR5-H3-23** | 0.458 | 0.042 | **10.90** |
| **CCR5-H3-26** | 0.638 | 0.048 | **13.29** |
| **CCR5-H3-43** | 1.533 | 0.037 | **41.43** |
| **CCR5-H3-44** | 1.303 | 0.111 | **11.74** |
| **CCR5-H4-1** | 0.889 | 0.079 | **11.25** |
| **CCR5-H4-13** | 0.792 | 0.028 | **28.29** |
| **CCR5-H4-18** | 2.116 | 0.024 | **88.17** |
| **CCR5-H4-30** | 1.871 | 0.141 | **13.27** |
| **CCR5-H4-34** | 1.346 | 0.042 | **32.05** |
| **CCR5-H4-35** | 1.989 | 0.229 | **8.69** |
| **CCR5-H4-36** | 1.994 | 0.128 | **15.58** |
| **CCR5-H4-40** | 0.728 | 0.103 | **7.07** |
| **CCR5-H4-61** | 2.069 | 0.134 | **15.44** |
| **CCR5-H4-79** | 1.807 | 0.138 | **13.09** |

^a^**Albumin,** as background control

^b^Defined as CCR5 peptide-Albumin divided by Albumin signal
